# Association of Instrumental Activities of Daily Living, Physical Function, and Mental Health among Older Returnees after the Fukushima Daiichi Nuclear Power Station Accident

**DOI:** 10.3390/ijerph182111639

**Published:** 2021-11-05

**Authors:** Nobuaki Moriyama, Yoshitaka Nishikawa, Wataru Hoshi, Tomomi Kuga, Hajime Iwasa, Tomoo Murayama, Tatsuya Itagaki, Yuta Saito, Seiji Yasumura

**Affiliations:** 1 Department of Public Health, Fukushima Medical University School of Medicine, Fukushima 960-1295, Japan; kuuga@fmu.ac.jp (T.K.); hajimei@fmu.ac.jp (H.I.); yasumura@fmu.ac.jp (S.Y.); 2 Kawauchi Village National Health Insurance Clinic, Kawauchi 979-1202, Japan; yoshitakanishikawa@gmail.com (Y.N.); qzb13723@nifty.com (T.M.); 3 Department of Internal Medicine, Hirata Central Hospital, Hirata 963-8202, Japan; 4 Department of Health Informatics, Kyoto University School of Public Health, Kyoto 606-8315, Japan; 5 Department of Rehabilitation, Hirata Central Hospital, Hirata 963-8202, Japan; watarururu0421@gmail.com (W.H.); ohmayoneeee@gmail.com (T.I.); yu.saito0603@gmail.com (Y.S.)

**Keywords:** instrumental activities of daily living, radiation accident, physical function, mental health, old returnees

## Abstract

The 2011 Great East Japan Earthquake and consequent Fukushima Daiichi Nuclear Power Station accident caused a large-scale evacuation, generating various health issues. Although residents gradually returned, their independence of daily living and associated factors remain unknown. This study examines the hypothesis that physical and mental status are associated with the instrumental activities of daily living (IADL) of older returnees (65 years and above) after the disaster. Older returnees to Kawauchi Village, Fukushima Prefecture, located 20–30 km southwest of the power plants, were recruited. IADL was assessed using the Japan Science and Technology Agency Index of Competence, physical function via the 30-s chair stand test, and mental health via the Japanese version of the World Health Organization Five Well-Being Index. To examine the association of IADL and possible factors, a t-test or Pearson’s product-moment correlation coefficient was used, stratified by sex. The data of 29 participants (75.5 ± 7.4 years, 19 female) were analyzed. Physical function was associated with IADL in females. Mental health was associated with IADL in males and females. Taking measures to strengthen physical function in females, as well as to improve mental health in both sexes, for enhancing IADL ability could be beneficial.

## 1. Introduction

Currently, the global trend is toward reduced fertility rates, increased life expectancy, and population aging. In Japan too, there has been a surge in the aging population: the proportion of older adults, expressed as the percentage of those aged 65 or older, reached 27.7% in 2017 [1]. Age is a major predictor of care needs [2] because of the inevitability of functional decline. Recently, the concept of healthy life expectancy, which is defined as the average number of years a person is expected to live without any limitations in daily activities [3], has been gathering attention [4]. The extension of healthy life expectancy is one of the targets of “Healthy Japan 21 (the second term)” promoted by the Japanese Ministry of Health, Labour and Welfare since 2013 [5]; the goal is to ensure that all citizens—from infants to older adults—have hope and live a meaningful life while supporting each other. Overall, the objective is to achieve a vibrant society that embraces healthy and spiritually rich lives according to life stage, along with a sustainable social security system.

Instrumental activities of daily living (IADL), originally advocated by Lawton and Brody [6], involve higher-level tasks than activities of daily living (ADL), such as financial and medication management, driving, shopping, house cleaning, and meal preparation. IADL abilities usually decline prior to ADL abilities [7] and have been shown to be a predictor of life expectancy [8] and frailty [9]. Since high IADL ability in older adults can increase their life expectancy, the maintenance of high IADL in this population is a significant public health goal.

IADL is relatively easily impaired at the time of disasters, one of the most noteworthy of which is the Great East Japan Earthquake (GEJE). This magnitude 9.0 earthquake, which occurred off the Pacific coast of Tohoku, Japan on 11 March 2011, was followed by a tsunami more than 13 m high, striking the Tokyo Electric Power Company’s Fukushima Daiichi Nuclear Power Station [10]. Resulting in the emission of radioactive materials into the air [11], the accident was graded level 7—the most serious—on the International Nuclear and Radiological Event Scale [12]. After the accident, an evacuation zone was designated by the national government. On March 11, an evacuation order was issued within a 3 km (km) radius of the power plants, and the order to stay indoors was issued within a 10 km radius. On 12 March, the evacuation order was expanded to within a 20 km radius. On 22 April, planned evacuation and emergency evacuation preparation zones were set outside a 20 km radius according to radiation dose [13].

Consequently, over 164,000 people in Fukushima were evacuated, whether forcefully or voluntarily [14]. This government order had an impact on the affected population’s lifestyles. It has been reported that older evacuees had reduced physical activity [15] and poor dietary intake [16]. Studies have suggested that changes in lifestyle, diet, exercise, and other personal habits trigger musculoskeletal pain and other health problems in evacuees [17].

Evacuation and relocation also affect evacuees’ mental health [18]. The proportion of individuals with psychological distress in the 2011 fiscal year was 14.6%, and the proportion remained higher than among the general population that was not affected by the GEJE [19]. Since inactivity and poor mental health are reported to be bidirectionally associated [20], mental health could impact evacuees’ active lifestyle and consequent physical functions.

After the evacuation order was lifted, some people decided to return home while others did not, thus continuing the separation of former residents. Following a disaster, the return of former residents is a crucial factor in the recovery of affected areas. Several key determinants affect evacuees’ decision to return to their hometowns, including a sense of attachment to their home, job obligations, presence of family members in the area [21], and a sense of anxiety regarding the risk and health effects of radiation exposure [22,23].

Furthermore, relocation could impact evacuees’ independence with regard to daily living. Inoue et al. [24] reported that the long-term care certification rate was higher in municipalities designated evacuation zones even six years after the GEJE. However, the long-term care utilization rate was lower in returnees compared with evacuees who had not returned, which could be because of limited resources or facilities for long-term care services in the evacuation zone [25]. According to the latest survey [26], those who had not yet decided whether to return home ranked “resumption of medical and nursing care facilities” as the most important condition for deciding to return. Thus, currently, possessing high IADL ability is a key factor in older evacuees’ decision to return.

A total of 35,137 individuals are still living as evacuees within or outside Fukushima Prefecture [27]. Some individuals plan to return to their hometowns after the evacuation order is lifted and once they feel ready to do so. To prevent a decline in IADL ability after returning home, healthcare providers working in local municipalities should make support plans so that they can maintain physical function and mental health. However, no study has examined independence in the lives of returnees and its association with their physical and mental status.

Thus, this study aimed to (1) describe the IADL ability of older returnees after the GEJE and compare it with the national norm and (2) examine the hypothesis that physical function and mental health are associated with IADL ability. The results of this study can provide physical therapists and other healthcare workers working in affected municipalities with data for effective practices to prevent impairments in IADL ability and subsequent long-term care following the radiation accident.

## 2. Materials and Methods

### 2.1. Design

This study employed a cross-sectional design. Data were collected from 2 to 13 November 2020. Although the participants live in a single municipality, owing to the large size of the village, repeatedly accessing the public health facility is not easy. Considering these circumstances, it was difficult to conduct a longitudinal investigation.

### 2.2. Setting

The study site was Kawauchi Village, Fukushima Prefecture, Japan. Kawauchi Village is located 20–30 km southwest of the power plants and is partially included in the Evacuation Order Area (within a 20 km radius of the plants). On 15 March 2011, the local government of Kawauchi Village decided to direct residents to evacuate, clearing almost the entire area. On 31 January 2012, the mayor of the village declared that residents could safely return to their homes because comparatively low levels of radiation were found [28]. As a result, residents gradually returned to the village, and in June 2016, all restrictions were lifted. At the time of the nuclear accident in 2011, approximately 3000 individuals lived in the village, and in September 2018, the population of the village was 2165 [29].

### 2.3. Participants

Older residents of Kawauchi Village were recruited for this study. Those aged 65 or older at the time of data collection were included. Individuals with impaired cognitive function exhibiting difficulty understanding the examiners’ instructions were excluded. Data were obtained at the public facility containing the health and welfare division of the village office of Kawauchi, a clinic, and a daycare center for seniors. Village residents were informed that a physical fitness test was to be held and related information, including venue and date, was widely distributed via flyers and public relations magazines in order to minimize deviation of participants’ basic characteristics. Data were collected from people who visited the venues for any reason, be it the physical fitness test or other work.

### 2.4. Measured Items

IADL were assessed using the Japan Science and Technology Agency Index of Competence (JST-IC) [30,31]. The JST-IC measures a level of daily function that is higher than ADL, including four domains: technology usage, information practice, life management, and social engagement. Each domain contains four items, and each item is scored on a dichotomous rating scale (0 = “no,” 1 = “yes”). The item scores are summed to obtain a total score (range, 0–16). Higher scores reflect a higher ability to perform IADL.

Physical function, mental health, and physical health status were assessed as potential factors associated with IADL. The 30-s (s) chair stand test [32] was administered to assess physical function. A registered public health nurse or physical therapist was responsible for the measurement. An assessor instructed the participant to (1) sit on a chair (40 cm in height, with a straight back without armrests) with their arms crossed in front of their chest and (2) keep their feet flat on the floor and back straight. The participant was encouraged to complete as many full stands as possible within 30 s. The participant was instructed to fully sit between each stand. The score was the total number of stands within 30 s.

The Japanese version of the World Health Organization Five Well-Being Index (WHO-5-J), a useful tool for measuring mental health in older Japanese community-dwelling adults [33], was used. The WHO-5-J measures participants’ mental health over two weeks through the following items: (1) felt cheerful and in good spirits, (2) felt calm and relaxed, (3) felt active and vigorous, (4) woke up feeling fresh and rested, and (5) daily life was filled with things that interested me. The response to each item is rated on a six-point scale from 0 to 5, with a possible maximum score of 25 points. A higher score indicates better mental health.

Physical health was assessed based on a history of chronic disease and physical pain. History of chronic disease was evaluated using the following question: “Have you ever been diagnosed with or received treatment for the following diseases: hypertension, diabetes, cerebral stroke, and heart disease?” The presence of physical pain was investigated using the following question: “Do you feel physical pain in your daily life?” Participants were dichotomized according to their responses of “yes” or “no” for each item.

In addition, sociodemographic characteristics including age, sex, economic circumstances (bad/a little bad/normal/moderate/good), employment status (employed/not employed), and frequency of outings (once per month or more/one to three times per month/seldom or never) were recorded.

### 2.5. Data Analysis

For the data analysis, descriptive data on IADL assessed using JST-IC scores were summarized and compared with the national normative data [31] in order to illustrate features of IADL ability in returnees. Thereafter, a *t*-test or Pearson’s product-moment correlation coefficient was used according to variable type to examine the association of IADL with physical health status, physical function, and mental health. Following a previous study’s suggestion that it is not the disease label but the symptoms of a disease that affect IADL [34], physical pain was considered representative of physical health status. In each univariate analysis, only data of individuals who provided two complete data were analyzed even though other items were incomplete. Data of individuals who did not complete the JST-IC were excluded from analysis. The analyses were then stratified according to sex. All data were analyzed using SPSS Statistics for Windows, version 21 (IBM Corp., Armonk, NY, USA).

### 2.6. Ethical Considerations

This study was approved by the Ethics Committee of Fukushima Medical University (approval number: General 2020-148). Written informed consent was obtained from all participants.

## 3. Results

Thirty-one individuals participated in this study. Two participants with missing IADL data were excluded from the analysis; thus, the data of 29 participants (75.5 ± 7.4 years, 10 males and 19 females) were analyzed (Table 1). We recruited all older returnees via a public relations magazine; the higher number of females in the sample was unintentional. Although we did not have the exact number of older adults residing in Kawauchi village at the time of measurement, the latest data show that the number was 838 as of September 2018. Therefore, approximately 3% of older returnees participated in this study.

The mean JST-IC score, which represented participants’ IADL, was 9.4 ± 3.0 (Table 2). In subgroups by sex, the mean score was 9.8 ± 3.0 in males and 9.2 ± 3.0 in females. The score with and without physical pain was 9.0 ± 3.5 and 10.6 ± 2.7, respectively, in males and 9.2 ± 3.2 and 9.3 ± 2.7, respectively, in females.

**Figure 1:**
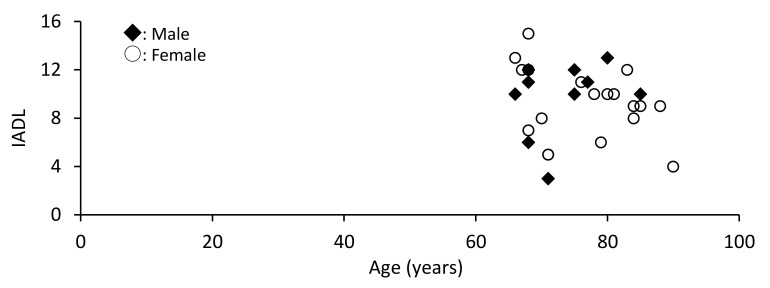
Association between age and instrumental activities of daily living (IADL) by sex.

**Figure 2:**
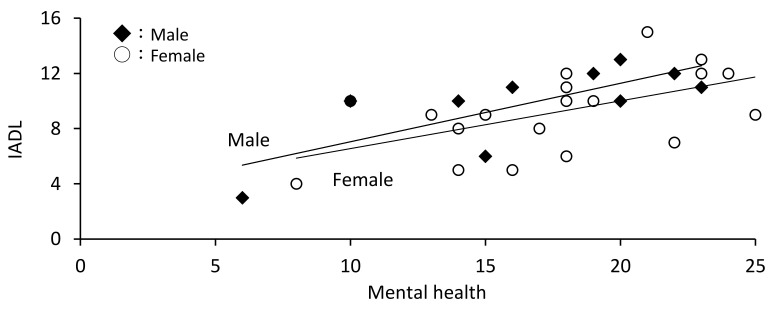
Association between mental health and instrumental activities of daily living (IADL) by sex.

**Figure 3:**
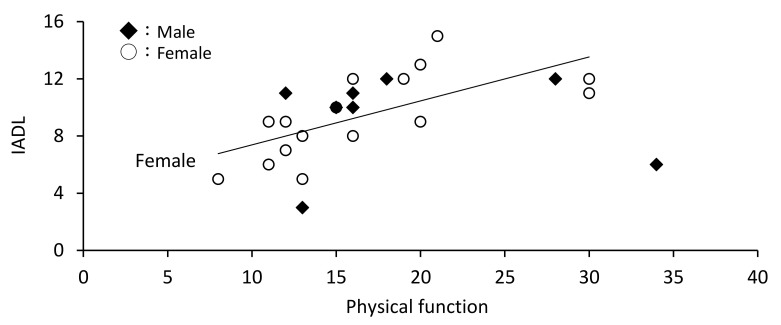
Association between physical function and instrumental activities of daily living (IADL) by sex.

In males, age and physical pain were not associated with IADL, whereas mental health was associated with IADL (*r* = 0.753, *p* = 0.012). Similarly, in females, age and physical pain were not associated with IADL, whereas physical function (*r* = 0.674, *p* = 0.004) and mental health (*r* = 0.548, *p* = 0.015) were associated with IADL (Table 3, Figure 1, Figure 2 and Figure 3).

## 4. Discussion

In this study, we examined the IADL of older residents in Kawauchi Village. There was no obvious difference compared with the national normative data (JST-IC score of 9.5 ± 4.2 vs. 9.4 ± 3.0 in the current study) [31] even though returnees were impacted by the disaster and experienced multiple relocations, suggesting that evacuation/relocation itself was not severely associated with their IADL abilities. In Kawauchi Village, there is a national health insurance clinic that contributes to healthcare service delivery [35]. Thus, returnees can easily manage their chronic diseases and physical pain, which, if ignored, could result in deteriorated physical health, directly impacting IADL ability.

Regarding physical function, the association with IADL was observed only in females. Previous studies have reported that muscle strength, assessed through grip strength, is associated with remaining independent in IADL in Japanese older adults [36,37,38]. Since muscle strength indicates basic physical function, the results of this study support previous findings.

Mental health was found to be associated with IADL ability in both males and females. Ormel et al. [39] also reported an association between IADL and depressive symptoms. Additionally, a prospective cohort study reported an association between psychological distress and the incident risk of functional disability among older survivors following the GEJE [40]. In adults who lived in evacuation zones, the proportion with psychological distress remained higher than among the general population [19]. Based on these findings, it is suggested that the maintenance of mental health is also significant for returnees. However, it should be noted that IADL disability and depressive symptoms mutually reinforce each other with time, suggesting the possibility of reverse causality [39].

Interestingly, age was not associated with IADL in either sex, contrary to national normative data [31]. This implies that aging was not directly associated with IADL; instead, factors that were affected by aging, such as functional decline, were directly associated with IADL. Therefore, measures should be focused on physical function and/or mental health, regardless of age.

To maintain IADL in returnees, the maintenance of mental health is important. It is hoped that individuals are supported in finding activities they can enjoy, such as having opportunities to interact with neighbors and engaging in hobbies. Matsuyama et al. [41] also reported that high individual-level and community-level social support were associated with low psychological distress in survivors of the GEJE. Based on this result, encouraging returnees to participate in various social gatherings (e.g., community exchanges among local residents) could be effective for maintaining their mental health. Amagasa et al. [42] reported that social participation has protective effects with regard to mental disorders, especially in women. For men, offering meaningful roles within organizations may be important for mental health [43]. Additionally, Komatsu et al. [44] reported that combined engagement in physical and cultural activities was associated with a lower risk of a subsequent decline in IADL.

Among older residents, for enhancing physical function, specific practices like possessing home equipment like an exercise mat or resistance band may be effective [45]. Greiner et al. [46] introduced an hour-long intervention program implemented once a week for 24 weeks in an area affected by the GEJE. The program comprised a 40 min exercise session and 20 min of social time, resulting in improved physical function in the participants. Another study reported that a group intervention involving physical activity improved mental health among older returnees after the disaster [47]. Such community-based activities might also be beneficial for promoting mental health among returnees.

The strength of this study lies in the fact that it is the first to clarify IADL and its associated factors in returnees at the time of data measurement in 2020: 9.5 years after the disaster in 2011 and four years after the lifting of all evacuation orders in 2016. However, it has some limitations. First, because of the cross-sectional design, causal relationships between IADL and associated factors could not be determined. Second, as the participants were not recruited by random sampling, they were not representative of all older returnees in Kawauchi Village. Since cognitive decline is reported to affect IADL [34,48], it is possible that IADL in the study population was overestimated. Third, the sample size was limited owing to restrictions on gatherings because of the coronavirus disease 2019 pandemic. Fourth, because of the low power of the statistical tests, it is possible that some correlates of IADL were missed. Fifth, as a multivariate analysis could not be conducted, the results of the univariate analyses without adjusting for covariates should be interpreted carefully. Finally, although cognitive function is reported to be associated with IADL [38], information regarding participants’ cognitive function was not collected; thus, the missing adjustment of cognitive function could have affected the results.

Despite these limitations, this study showed the status of IADL in areas affected by the nuclear accident. Healthcare professionals and government staff in charge of health promotion in older residents should consider some measures to improve residents’ mental health along with maintaining physical function, especially in females.

## 5. Conclusions

In comparison to national normative data, IADL ability in older residents who returned to a former evacuation area had not noticeably deteriorated. Physical function in females and mental health in both sexes were associated with the ability to perform IADL.

To maintain returnees’ IADL ability, improving physical function via encouraging active lifestyles in females and mental health via providing opportunities to meet other local residents could be beneficial. Further studies with larger sample sizes and a longitudinal design are necessary to clarify the determinants of IADL in the target population.

## Figures and Tables

**Table 1:** Participants’ characteristics.

Item	Categories	*n* or Mean ± Standard Deviation
Age		75.5 ± 7.4
Sex	Male	10
	Female	19
Economic circumstance	Bad	0
	A little bad	1
	Normal	25
	Moderate	3
	Good	0
Chronic disease history	Present/Absent	19/10
Physical pain	Present/Absent	18/11
Employment status	Employed/Not employed	6/23
Frequency of outings	Once per month or more	26
	One to three times per month	3
	Seldom or never	0
Mental health *^1^		17.3 ± 4.9
Functional ability *^2^		17.4 ± 6.7

*^1^ Score of World Health Organization Five Well-Being Index. *^2^ Score of 30-s chair stand test.

**Table 2:** JST-IC scores as representative of IADL.

	Total (*n* = 29)	Males (*n* = 10)	Females (*n* = 19)
Technology usage	2.2 ± 1.3	2.8 ± 1.3	1.9 ± 1.3
Information practice	2.7 ± 1.0	2.4 ± 1.2	2.8 ± 1.0
Life management	2.5 ± 0.9	2.5 ± 1.0	2.5 ± 1.0
Social engagement	2.0 ± 1.5	1.8 ± 1.5	2.1 ± 1.4
Total	9.4 ± 3.0	9.8 ± 3.0	9.2 ± 3.0

Mean ± Standard Deviation. JST-IC: Japan Science and Technology Agency Index of Competence; IADL: instrumental activities of daily living.

**Table 3:** Correlation between IADL and possible associated factors (stratified by sex).

	Males (n = 10)		Females (n = 19)	
	r (95%CI)	*p*	r (95%CI)	*p*
Age	0.287 (–0.418–0.776)	0.422	–0.324 (–0.678–0.153)	0.176
Mental health *^1^	0.753 (0.235–0.938)	0.012	–0.548 (0.125–0.803)	0.015
Physical function *^2^	–0.080 (–0.676–0.579)	0.838	–0.674 (0.317–0.864)	0.004

*^1^ Score of World Health Organization Five Well-Being Index. *^2^ Score of 30-s chair stand test. IADL: instrumental activities of daily living; CI: confidence interval.
